# Evidence of Functional Protein Dynamics from X-Ray Crystallographic Ensembles

**DOI:** 10.1371/journal.pcbi.1000911

**Published:** 2010-08-26

**Authors:** Jonathan E. Kohn, Pavel V. Afonine, Jory Z. Ruscio, Paul D. Adams, Teresa Head-Gordon

**Affiliations:** 1 Department of Bioengineering, University of California, Berkeley, Berkeley, California, United States of America; 2 Physical Biosciences Division, Lawrence Berkeley National Laboratory, Berkeley, California, United States of America; Stanford University, United States of America

## Abstract

It is widely recognized that representing a protein as a single static conformation is inadequate to describe the dynamics essential to the performance of its biological function. We contrast the amino acid displacements below and above the protein dynamical transition temperature, T_D_∼215K, of hen egg white lysozyme using X-ray crystallography ensembles that are analyzed by molecular dynamics simulations as a function of temperature. We show that measuring structural variations across an ensemble of X-ray derived models captures the activation of conformational states that are of functional importance just above T_D_, and they remain virtually identical to structural motions measured at 300K. Our results highlight the ability to observe functional structural variations across an ensemble of X-ray crystallographic data, and that residue fluctuations measured in MD simulations at room temperature are in quantitative agreement with the experimental observable.

## Author Summary

There is a well-recognized gap between the dynamical motions of proteins required to execute function and the experimental techniques capable of capturing that motion at the atomic level. We show that much experimental detail of dynamical motion is already present in X-ray crystallographic data, which arises from being solved by different research groups using different methodologies under different crystallization conditions, which then capture an ensemble of structures whose variations can be quantified on a residue-by-residue level using local density correlations. We contrast the amino acid displacements below and above the protein dynamical transition temperature, T_D_∼215K, of hen egg white lysozyme by comparing the X-ray ensemble to MD ensembles as a function of temperature. We show that measuring structural variations across an ensemble of X-ray derived models captures the activation of conformational states that are of functional importance just above T_D_ and they remain virtually identical to structural motions measured at 300K. It provides a novel analysis of large X-ray ensemble data that is useful for the broader structural biology community.

## Introduction

It has been suggested that at temperatures below the protein dynamical transition temperature, T_D_, there is a dominant native basin in which a protein's dynamics is largely controlled by harmonic motions [1]. Above this temperature, a sudden activation of new anharmonic protein motions that are thought to be dependent on a more fluid solvent environment [2], correlates with a rapid enhancement of enzymatic function in most cases. The importance of dynamics in mediating protein function is widely recognized [3], and experimental techniques such as nuclear magnetic resonance (NMR), quasi-elastic neutron scattering, dielectric relaxation, Mossbauer and terahertz time domain spectroscopies have been used to explore the dynamical transition behavior of proteins with temperature and water solvent [4], [5], [6], [7], [8], [9], [10], [11], [12]. Much evidence supports the idea that activation of solvent dynamics must proceed first in order to initiate the dynamical transition to a functional protein, emphasizing that the protein itself plays a more passive role in the concept known as “solvent slaving”. [13]

Because the majority of X-ray crystal structures of proteins are modeled as single conformations [14] in crystalline environments, their dynamical information is limited. Dynamics is often indirectly addressed by theoretical estimates of uncertainty in atomic positions using Luzzati or Read plots [15], [16] and isotropic or anisotropic B-factors that measure primarily molecular disorder in the crystal, and possibly other errors, in addition to thermal motion [17], [18]. More recently, an increasing number of high-resolution data sets have permitted the anisotropic refinement of disordered regions and the modeling of alternate backbone and side-chain conformations [19], [20], although most protein crystals diffract to too low resolution for the modeling of disorder in general [21]. Some limited information on conformational mobility can be determined from multi-start simulated annealing refinement [22], [23], multi-copy refinement [24], or time-averaging with multiple refinements [25], [26], [27], producing different structures because each instance fits the structure factor data slightly differently. Furthermore, given the high amount of automation in modern day protein crystallography, the entire procedure of model building, density modification and refinement can yield an ensemble of structures compatible with a given X-ray data set.[28]

In this work we define several experimental ensembles with respect to the X-ray crystallography derived hen egg white lysozyme (HEWL) structure 3LZT [29] which are analyzed by comparing them to MD generated ensembles at different temperatures. HEWL is unique among almost all proteins in the PDB because (1) there is at least one high resolution structure that serves as the reference (here 3LZT), (2) it contains no prosthetic groups or metals, (3) it is a protein that has been solved in multiple space groups, and (4) there are ∼80 independent solved structures to generate an experimental ensemble. Surprisingly virtually no other protein allows us to do the same analysis under these criteria. Based on this data, the experimental ensembles include: *(1)* the X-ray ensemble generated from multi-start simulated annealing of 3LZT (3LZT-MSSA), *(2)* the X-ray ensemble of HEWL structures that crystallize in the P1 space group like 3LZT, and *(3)* the X-ray ensemble of HEWL structures that crystallizes into alternative space groups to P1, which we refer to as the non-P1 ensemble. These experimental ensembles, whose structure factor data was generated over the period between 1974 and 2010 (see supplementary material), are compared to MD simulations of HEWL in water, employing 3LZT as an initial configuration, and simulated at temperatures of 200K, 210K, 220K, 230K, and 300K.

## Methods

The HEWL protein (3LZT) was simulated in the AMBER9 [30] molecular mechanics package using the AMBER99SB (protein) [31] and TIP4P-Ew (water) [32] force fields. The HEWL protein was immersed in a box of 5736 water molecules and 9 Cl- and equilibrated by first restraining the protein atoms with a 10 kcal/mol/Å^2^ restraint while the system was heated from 0 to 200K, 210K, 220K, 230K or 300K using the Andersen thermostat under constant volume conditions. After equilibration, the system underwent 10ns of NPT molecular dynamics, sufficient to generate a stable protein within the MD model whose RMSD does not vary after the first 1ns, consistent with previous studies [33], [34]. The equations of motion are integrated with 1fs timesteps, the long-range electrostatic interactions are calculated using Particle Mesh Ewald method, and a cutoff of 10.0Å is used for real space electrostatics and Lennard-Jones interactions. All bonds involving hydrogens were constrained using the SHAKE algorithm. The system was then equilibrated under constant pressure using the AMBER default Berendsen barostat parameters at 1atm for 1 ns. The molecular dynamics ensembles at each temperature were derived from 100 snapshots separated by 0.01ns over each 10ns trajectory for each temperature.

All crystallographic analysis was performed using the program PHENIX [35]. For consistency the original HEWL model (3LZT) used throughout this study was re-refined in *phenix.refine* [36] at 1.10Å resolution, yielding a high quality structure with an R-factor = 0.0990 and R-free = 0.1308. [37] We then performed multi-start simulated annealing refinement against the high resolution data set of 3LZT to generate the 3LZT_MSSA reference ensemble. All X-ray ensembles and molecular dynamics structures at different temperatures were superimposed against the refined 3LZT model using *phenix.superpose_pdbs*, and using *phenix.model_vs_data*, and all resultant R-factors and RMSDs recorded (see selection reported in Table 1). Snapshots sampled from the MD trajectory at each temperature were least square fit to the experimental real space data of 3LZT and B-factors set to an average value. Structure factor data was then calculated *but without atomic relaxation of atomic positions*, in order to compare the structural deviations of the MD model from the 3LZT X-ray structure reference.

**Table 1:** R-factor and RMSD (computed using Cα-atoms only) with respect to the 3LZT reference for the experimental X-ray ensembles and the molecular dynamics ensembles at two different temperatures.

Experimental Ensembles	R-work	RMSD (Å)
**3LZT-MSSA**	0.1794	0.07
**P1 Lysozymes**	0.3422	0.33
**Non-P1 Lysozymes**	0.4320	0.75

Simulated Ensembles	R-work	RMSD (Å)
**200K-MD: 3LZT**	0. 4427	0.58
**300K-MD: 3LZT**	0. 4635	1.00

See Table S1 for X-ray structures used.

We quantify the structural variations on a per residue basis among the ensembles by calculating the local density correlation (LDC) coefficients of electron density values between X-ray and MD ensembles computed from the model maps around individual amino-acid residues [38], [39], [40]

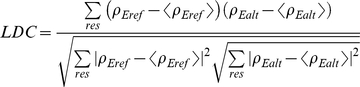
(1)where the ρ's are electron density values at grid points, and *Eref* and *Ealt* refer to densities of the members of the reference 3LZT-MSSA ensemble and the alternative ensemble to be compared, respectively. Computing a LDC requires two maps, and each map can be computed from one single model or an ensemble of models (for example, a PDB file containing multiple HEWL models in the P1 space group split by MODEL-ENDMDL records).

Residue level LDCs between the ensembles of structures were computed using *phenix.real_space_correlation* tool. Computing a LDC for a residue (for example) requires defining a region around a residue in both maps and the grid points in those defined regions are then used in the LDC calculation. The region around a residue can be defined assuming that each atom has radius of 1.5–2.0Å. Since the LDC does not depend on the scale the occupancies of atoms in ensemble containing N models do not need to be divided by N. We categorize LDC values between structural ensembles greater than 0.7 as having a strong correlation [41], [42].

## Results

**Figure 1:**
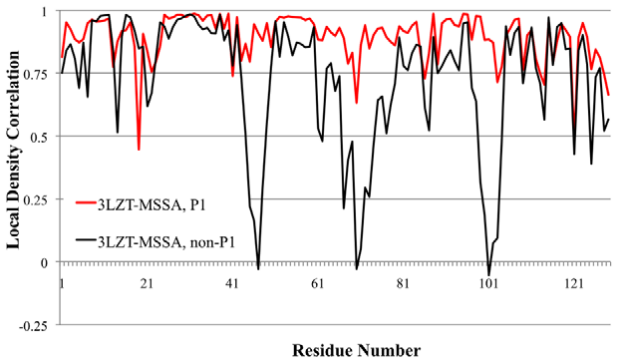
Local density correlations for P1 and non-P1 X-ray ensembles. The P1 space group ensemble and the non-P1 space group ensembles compared to the 3LZT-MSSA ensemble. This defines the experimentally allowed regions of disorder on a residue-by-residue basis. The largest deviations measured between the P1 and non-P1 ensemble captures the functional motions corresponding to the β-turn connecting the first two strands of the β-sheet (residues 44–50) and the central portion of long loop (residues 67–73) in the β-domain, and the enhanced fluctuations in the N-terminus and C-terminus in the α-domain, around a central hinge [47].

Figure 1 shows a comparison of the LDCs between the P1 and non-P1 X-ray ensembles for HEWL against the MSSA-3LZT reference ensemble. First it is noteworthy that >90% of residues of the P1 ensemble are well correlated with the 3LZT-MSSA reference, although there are deviations in LDC<0.7 in a few isolated regions. More interesting is the greater dissimilarity between the alternative crystal space groups of the non-P1 ensemble, which shows many regions that are poorly correlated with the 3LZT-MSSA and P1 ensembles. It is important to emphasize that if a member of the non-P1 HEWL ensemble had been chosen as a MSSA reference, the same regions of difference would be found for the P1 ensembles since the LDC analysis is symmetric between opposite definition of the reference ensemble.

The LDC deviations seen between the experimental X-ray ensembles show remarkable correlation with NMR S^2^ order parameters for backbone amide groups measured in ^15^N relaxation experiments [43], [44]. Although NMR order parameters have been compared to atomic B-factors of HEWL X-ray structures previously [43], [45], we have used the LDC and a far larger experimental ensemble of ∼80 different HEWL structure that shows far better quantitative agreement than previously described. Regions of S^2^<0.8 for HEWL correspond to residues 16–19, 45–50, 67–70, 116–119, while even lower S^2^ values were measured for residues 85–86 (loop preceding the C-helix), 102–106 (the loop connecting the C-helix and D-helix in the α-domain), and residues 127–129 in the C-terminus[43]. Previous normal mode analysis of HEWL has shown that the lowest frequency mode [45], a strong mechanistic indicator of protein function [46], corresponds to activation of the β-turn connecting the first two strands of the β-sheet (residues 44–50) and the central portion of long loop (residues 67–73) in the β-domain, and the enhanced fluctuations in the N-terminus (1–39) and C-terminus (116–129) in the α-domain, around a central hinge [47]. It is evident that the X-ray ensembles exhibiting regions of LDC<0.7, captures the NMR disorder of an aqueous thermal environment and normal mode analysis relevant for HEW lysozyme function quite well. Thus the experimental X-ray ensemble can measure the activation of functional motions of the protein at a residue-by-residue level as we compare to LDCs of molecular dynamics simulations below and above T_D_∼215K.

**Figure 2:**
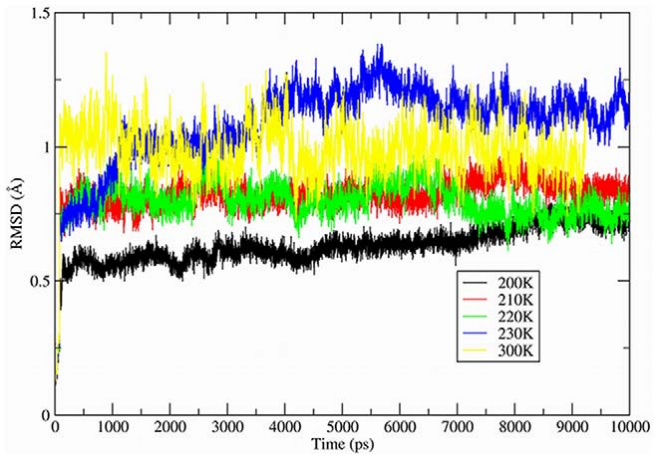
Molecular dynamics trajectory data of RMSD (against the 3LZT reference) at different temperatures. 200K (black), 210K (red), 220K (green), 230K (blue) and 300K (yellow).

**Figure 3:**
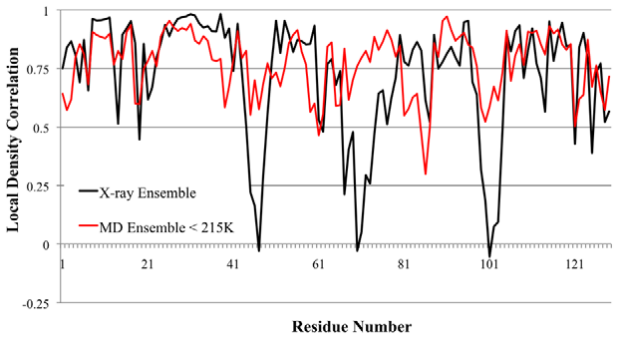
Local density correlations for X-ray ensemble and MD ensemble <215K. The values of LDC<0.75 seen in the X-ray ensemble measures captures the functional motions of the protein at a residue level (see text and Figure 1). It is apparent that below 215K, no functional motions have been activated.

Figure 2 shows the time progression of the root-mean-square deviation (RMSD) of the molecular dynamics trajectory from the 3LZT start state at each temperature. It is evident that the simulation model shows that a structural transition has occurred over the temperature range of 210K–230K. Figure 3 shows the LDC's for the experimental ensemble against the averaged MD ensembles at 200K and 210K (which were found to be very similar to each other and hence we averaged their LDC ensemble data). Below the transition temperature of ∼215K, a majority of residues (92 out of 129) are highly similar (LDC>0.7) to the 3LZT reference, while 19 of the 37 residues with an LDC<0.7 are within experimental deviations permitted under different crystallization conditions. For the remaining 18 residues outside of experimental differences, ∼11 residues have slightly degraded LDC values ranging from 0.6 to 0.7, with the remaining larger differences outside of experiment isolated to residues 80–86. Nonetheless, the overall dynamical motions of the aqueous solution of HEW lysozyme below 215K are not activated in any of the highly flexible or global motion regions that signify the active state of the protein.

**Figure 4:**
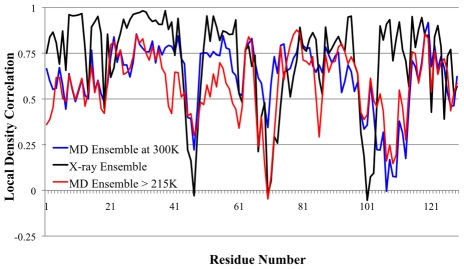
Local density correlations for X-ray ensemble and MD ensemble >215K and at 300K. The values of LDC<0.75 seen in the X-ray ensemble measures captures the functional motions of the protein at a residue level (see text and Figure 1). It is apparent that above 215K, the functional motions have been activated.

Figure 4 shows the LDC's for the experimental ensemble against the averaged MD ensembles at 220K and 230K (which were found to be very similar to each other and hence we averaged their ensemble data). We note in the region around residues 97 to 105 the X-ray ensemble shows very low LDC values, while this LDC minimum is broader over the residue range from 97 to 114 for the MD ensembles. This is because the triclinic and tetragonal crystal forms that dominate our experimental X-ray ensemble have a large number of atomic crystal contacts in the region of 105 to 114, suppressing their fluctuations. This suggests that our X-ray ensemble is incomplete, and we predict that a different crystal form of HEWL that relieves those contacts would bear out the MD fluctuations in this small region. Nonetheless, above the transition temperature of ∼215K, a *majority* of residues (90 out of 129) are now dissimilar (LDC<0.7) to the 3LZT reference, with 40 of the 90 dissimilar residues yielding LDC values less than 0.5 in the same regions as the overall X-ray ensemble. This is due to activation of global motions of the α- and β-domains about the central hinge, signifying that fluctuations of an active protein are now populated. Figure 3 also shows that the MD ensemble just above T_D_ is measuring structural deviations that are mostly identical to the MD ensemble at 300K, thereby showing that the functional dynamical signatures are largely complete just past the protein dynamical transition temperature.

## Discussion

It has been suggested that at temperatures below the protein dynamical transition temperature there is a dominant native basin in which a protein's dynamics is largely controlled by harmonic motions [1], and only upon activation of anharmonic motions above the dynamical transition temperature is a protein capable of executing its function. In turn, the temperature-dependent activation of protein flexibility has been shown to be controlled by the dynamical processes of the aqueous solvent environment [48], [49], i.e. the rigidity of solvent below T_D_ and the abrupt increase in dynamical plasticity of the water network above T_D_. Similarly, the effect of crystal packing forces is to reduce the mobility of the protein in the regions of crystal contacts [50], although the residue-specific regions of reduced mobility will change under crystallization into different crystal space groups. We have shown that a large ensemble of X-ray measurements taken in a number of different crystal space groups and solvent conditions are able to capture nearly the full range of amino acid fluctuations that are of functional importance. Our MD ensembles of HEW lysozyme, which take into account the temperature dependence of the solvent dynamics, show that just above T_D_∼215K the relevant fluctuations become fully populated and are largely equivalent to that observed at room temperature.

Previous studies have taken advantage of MD to characterize dynamical signatures present in experimental X-ray data [51], [52], but just as relevant is whether the conformational states sampled during the simulation at room temperature remain consistent with the X-ray model. During the time course of a MD simulation the structures of simulated proteins necessarily drift from the initial PDB coordinates due to thermal motion and a fluid environment that is different from the crystalline state. At the same time, the fluctuations away from the X-ray start state during the computed MD trajectory may mask the possibility that the parameters of the model force field are inadequate, and potentially giving misleading information on functional conformational states. This work shows that residue fluctuations measured in MD at room temperature are completely consistent with the structural deviations measured in the experimental X-ray ensembles. This is consistent with one of the main result in protein structure prediction in that physical energy functions are quite robust in ranking X-ray crystallography structures as lower in energy than non-native decoys [53], [54] and can successfully interrogate active site dynamics [55]. This is a mutually reinforcing result in the sense that the artificial crystalline environment is not problematic since the X-ray native basin holds under the fluid aqueous environment simulated in the MD trajectory.

### Conclusions

It is widely recognized that representing a protein as a single static conformation is inadequate to describe the dynamics essential to the performance of its biological function. X-ray crystal structures have historically relied on atomic displacement parameters and similar metrics to provide information on local flexibility and disorder [16], [17], but more recently have included multiple models consistent with a given set of structure factor data to better represent the dynamical ensemble [14], [28]. However the possibility of generating structure factor data for a given protein in different crystal forms and solvent conditions could generate an ensemble of structures that reveal the functionally relevant protein conformational states that are populated under physiological conditions.

## Supporting Information

Table S1 Supporting material.
(0.39 MB DOC)
