# Supplementary material for: Evidence of Functional Protein Dynamics from X-Ray Crystallographic Ensembles
Source: PLoS Comput Biol. 2010 Aug 26;6(8):e1000911. doi: 10.1371/journal.pcbi.1000911 (PMC2928775; doi:10.1371/journal.pcbi.1000911)
Supplement: Table S1 — Supporting material. (0.39 MB DOC) [file pcbi.1000911.s001.doc]

**Table S1.** Experimental X-ray structures employed in local density correlation and R-factor calculations.

| **PDB** | **Space Group** | **Reference** |
| --- | --- | --- |
| 1LZT | P1 | (Hodsdon et al., 1990) |
| 1V7S | P1 | (Harata and Akiba, 2006) |
| 2LZT | P1 | (Ramanadham et al., 1990) |
| 2VB1 | P1 | (Wang et al., 2007) |
| 4LZT | P1 | (Walsh et al., 1998) |
| 1LMA | P12_1_1 | (Madhusudan et al., 1993) |
| 1XEI | P12_1_1 | (Nagendra et al., 1998) |
| 1XEJ | P12_1_1 | (Nagendra et al., 1998) |
| 1XEK | P12_1_1 | (Nagendra et al., 1998) |
| 2D4L | P12_1_1 | (Harata and Akiba, 2006) |
| 1AKI | P2_1_2_1_2_1_ | (Artymiuk et al., 1982) |
| 1BGI | P2_1_2_1_2_1_ | (Oki et al., 1999) |
| 1F0W | P2_1_2_1_2_1_ | (Biswal et al., 2000) |
| 1F10 | P2_1_2_1_2_1_ | (Biswal et al., 2000) |
| 1HSX | P2_1_2_1_2_1_ | (Madhusudan et al., 1993) |
| 1JJI | P2_1_2_1_2_1_ | (Datta et al., 2001) |
| 1JPO | P2_1_2_1_2_1_ | (Bradbrook et al., 1995) |
| 1VDQ | P2_1_2_1_2_1_ | (Aibara et al., To be published-b) |
| 1VED | P2_1_2_1_2_1_ | (Aibara et al., To be published-b) |
| 2ZQ3 | P2_1_2_1_2_1_ | (Aibara et al., To be published-b) |
| 2ZQ4 | P2_1_2_1_2_1_ | (Aibara et al., To be published-b) |
| 193L | P4_3_2_1_2 | (Vaney et al., 1996) |
| 194L | P4_3_2_1_2 | (Vaney et al., 1996) |
| 1AZ4 | P4_3_2_1_2 | (Lim et al., 1998) |
| 1B0D | P4_3_2_1_2 | (Vaney et al., 2001) |
| 1BHZ | P4_3_2_1_2 | (Ramin et al., 1999) |
| 1BVX | P4_3_2_1_2 | (Dong et al., 1999) |
| 1BWH | P4_3_2_1_2 | (Dong et al., 1999) |
| 1BWI | P4_3_2_1_2 | (Dong et al., 1999) |
| 1BWJ | P4_3_2_1_2 | (Dong et al., 1999) |
| 1C10 | P4_3_2_1_2 | (Prange et al., 1998) |
| 1DPW | P4_3_2_1_2 | (Weiss et al., 2000) |
| 1DPX | P4_3_2_1_2 | (Weiss et al., 2000) |
| 1GWD | P4_3_2_1_2 | (Evans and Bricogne, 2002) |
| 1H87 | P4_3_2_1_2 | (Girard et al., 2002) |
| 1IEE | P4_3_2_1_2 | (Sauter et al., 2001) |
| 1JIS | P4_3_2_1_2 | (Datta et al., 2001) |
| 1JIT | P4_3_2_1_2 | (Datta et al., 2001) |
| 1JIY | P4_3_2_1_2 | (Datta et al., 2001) |
| 1LSA | P4_3_2_1_2 | (Kurinov and Harrison, 1995) |
| 1LSB | P4_3_2_1_2 | (Kurinov and Harrison, 1995) |
| 1LSC | P4_3_2_1_2 | (Kurinov and Harrison, 1995) |
| 1LSE | P4_3_2_1_2 | (Kurinov and Harrison, 1995) |
| 1LSF | P4_3_2_1_2 | (Kurinov and Harrison, 1995) |
| 1LYZ | P4_3_2_1_2 | (Diamond, 1974) |
| 1N4F | P4_3_2_1_2 | (Retailleau and Prange, 2003) |
| 1VAT | P4_3_2_1_2 | (Takeda et al., 2004) |
| 1VAU | P4_3_2_1_2 | (Takeda et al., 2004) |
| 1VDS | P4_3_2_1_2 | (Aibara et al., To be published-c) |
| 1VDT | P4_3_2_1_2 | (Aibara et al., To be published-c) |
| 1W6Z | P4_3_2_1_2 | (Jakoncic et al., 2006) |
| 1YI0 | P4_3_2_1_2 | (Deshpande et al., 2005) |
| 1YIK | P4_3_2_1_2 | (Hunter et al., 2005) |
| 1YIL | P4_3_2_1_2 | (Hunter et al., 2005) |
| 1YKZ | P4_3_2_1_2 | (Deshpande et al., 2005) |
| 1YL1 | P4_3_2_1_2 | (Deshpande et al., 2005) |
| 1Z55 | P4_3_2_1_2 | (Deshpande et al., 2005) |
| 2A6U | P4_3_2_1_2 | (Basso et al., 2005) |
| 2BLX | P4_3_2_1_2 | (Nanao et al., 2005) |
| 2C8O | P4_3_2_1_2 | (Vernede et al., 2006) |
| 2C8P | P4_3_2_1_2 | (Vernede et al., 2006) |
| 2CGI | P4_3_2_1_2 | (Jakoncic et al., 2006) |
| 2EPE | P4_3_2_1_2 | (Naresh et al., To be published) |
| 2H9J | P4_3_2_1_2 | (Hunter et al., 2007) |
| 2H9K | P4_3_2_1_2 | (Hunter et al., 2007) |
| 2HU1 | P4_3_2_1_2 | (Wine et al., 2007) |
| 2HUB | P4_3_2_1_2 | (Frolow et al., To be published) |
| 2LYM | P4_3_2_1_2 | (Kundrot and Richards, 1987a) |
| 2LYZ | P4_3_2_1_2 | (Diamond, 1974) |
| 2YVB | P4_3_2_1_2 | (Naresh et al., To be Published) |
| 3E3D | P4_3_2_1_2 | (Beck et al., 2008) |
| 3EXD | P4_3_2_1_2 | (Guimaraes et al., 2009) |
| 3LYM | P4_3_2_1_2 | (Kundrot and Richards, 1987b) |
| 3LYZ | P4_3_2_1_2 | (Diamond, 1974) |
| 4LYM | P4_3_2_1_2 | (Kodandapani et al., 1990) |
| 4LYZ | P4_3_2_1_2 | (Diamond, 1974) |
| 5LYT | P4_3_2_1_2 | (Young et al., 1993) |
| 5LYZ | P4_3_2_1_2 | (Diamond, 1974) |
| 6LYT | P4_3_2_1_2 | (Beddell et al., 1975) |
| 6LYZ | P4_3_2_1_2 | (Diamond, 1974) |

Aibara, S., Suzuki, A., Kidera, A., Shibata, K., Yamane, T., Delucas, L.J., and Hirose, M. (To be published-a). The crystal structure of the monoclinic form of hen egg white lysozyme at 1.7 angstroms resolution in space.

Aibara, S., Suzuki, A., Kidera, A., Shibata, K., Yamane, T., Delucas, L.J., and Hirose, M. (To be published-b). The crystal structure of the orthorhombic form of hen egg white lysozyme at 1.5 angstroms resolution.

Aibara, S., Suzuki, A., Kidera, A., Shibata, K., Yamane, T., Delucas, L.J., and Hirose, M. (To be published-c). The crystal structure of the tetragonal form of hen egg white lysozyme at 1.6 angstroms resolution in space.

Artymiuk, P.J., Blake, C.C.F., Rice, D.W., and Wilson, K.S. (1982). The Structures of the Monoclinic and Orthorhombic Forms of Hen Egg-White Lysozyme at 6-a Resolution. Acta Crystallogr B *38*, 778-783.

Basso, S., Fitch, A.N., Fox, G.C., Margiolaki, I., and Wright, J.P. (2005). High-throughput phase-diagram mapping via powder diffraction: a case study of HEWL versus pH. Acta Crystallogr D *61*, 1612-1625.

Beck, T., Krasauskas, A., Gruene, T., and Sheldrick, G.M. (2008). A magic triangle for experimental phasing of macromolecules. Acta Crystallogr D *64*, 1179-1182.

Beddell, C.R., Blake, C.C.F., and Oatley, S.J. (1975). An X-Ray Study of Structure and Binding Properties of Iodine-Inactivated Lysozyme. J Mol Biol *97*, 643-&.

Biswal, B.K., Sukumar, N., and Vijayan, M. (2000). Hydration, mobility and accessibility of lysozyme: structures of a pH 6.5 orthorhombic form and its low-humidity variant and a comparative study involving 20 crystallographically independent molecules. Acta Crystallogr D *56*, 1110-1119.

Bradbrook, G.M., Helliwell, J.R., and Habash, J. (1995). Low temperature orthorhombic lysozyme. Proc.Spie-Int.Soc.Opt.Eng. *2521*, 160.

Brinkmann, C., Weiss, M.S., and Weckert, E. (2006). The structure of the hexagonal crystal form of hen egg-white lysozyme. Acta Crystallogr D *62*, 349-355.

Datta, S., Biswal, B.K., and Vijayan, M. (2001). The effect of stabilizing additives on the structure and hydration of proteins: a study involving tetragonal lysozyme. Acta Crystallogr D *57*, 1614-1620.

Deshpande, A., Nimsadkar, S., and Mande, S.C. (2005). Effect of alcohols on protein hydration: crystallographic analysis of hen egg-white lysozyme in the presence of alcohols. Acta Crystallogr D *61*, 1005-1008.

Diamond, R. (1974). Real-Space Refinement of Structure of Hen Egg-White Lysozyme. J Mol Biol *82*, 371-&.

Dong, J., Boggon, T.J., Chayen, N.E., Raftery, J., Bi, R.C., and Helliwell, J.R. (1999). Bound-solvent structures for microgravity-, ground control-, gel- and microbatch-grown hen egg-white lysozyme crystals at 1.8 angstrom resolution. Acta Crystallogr D *55*, 745-752.

Evans, G., and Bricogne, G. (2002). Triiodide derivatization and combinatorial counter-ion replacement: two methods for enhancing phasing signal using laboratory Cu K alpha X-ray equipment. Acta Crystallogr D *58*, 976-991.

Frolow, F., Lagziel-Simis, S., Cohen-Hadar, N., Wine, Y., and Freeman, A. (To be published). Monitoring influence of pH on the molecular and crystal structure of hen egg-white tetragonal lysozyme.

Girard, E., Chantalat, L., Vicat, J., and Kahn, R. (2002). Gd-HPDO3A, a complex to obtain high-phasing-power heavy-atom derivatives for SAD and MAD experiments: results with tetragonal hen egg-white lysozyme. Acta Crystallogr D *58*, 1-9.

Guimaraes, B.G., Sanfelici, L., Neuenschwander, R.T., Rodrigues, F., Grizolli, W.C., Raulik, M.A., Piton, J.R., Meyer, B.C., Nascimento, A.S., and Polikarpov, I. (2009). The MX2 macromolecular crystallography beamline: a wiggler X-ray source at the LNLS. J Synchrotron Radiat *16*, 69-75.

Harata, K. (1994). X-Ray Structure of a Monoclinic Form of Hen Egg-White Lysozyme Crystallized at 313-K - Comparison of 2 Independent Molecules. Acta Crystallogr D *50*, 250-257.

Harata, K., and Akiba, T. (2006). Structural phase transition of monoclinic crystals of hen egg-white lysozyme. Acta Crystallogr D *62*, 375-382.

Herzberg, O., and Sussman, J.L. (1983). Protein Model-Building by the Use of a Constrained-Restrained Least-Squares Procedure. J Appl Crystallogr *16*, 144-150.

Hodsdon, J.M., Brown, G.M., Sieker, L.C., and Jensen, L.H. (1990). Refinement of Triclinic Lysozyme .1. Fourier and Least-Squares Methods. Acta Crystallogr B *46*, 54-62.

Hunter, T.M., McNae, I.W., Liang, X.Y., Bella, J., Parsons, S., Walkinshaw, M.D., and Sadler, P.J. (2005). Protein recognition of macrocycles: Binding of anti-HIV metallocyclams to lysozyme. P Natl Acad Sci USA *102*, 2288-2292.

Hunter, T.M., Mcnae, I.W., Simpson, D.P., Smith, A.M., Moggach, S., White, F., Walkinshaw, M.D., Parsons, S., and Sadler, P.J. (2007). Configurations of nickel-cyclam antiviral complexes and protein recognition. Chem-Eur J *13*, 40-50.

Jakoncic, J., Di Michiel, M., Zhong, Z., Honkimaki, V., Jouanneau, Y., and Stojanoff, V. (2006). Anomalous diffraction at ultra-high energy for protein crystallography. J Appl Crystallogr *39*, 831-841.

Kodandapani, R., Suresh, C.G., and Vijayan, M. (1990). Crystal-Structure of Low Humidity Tetragonal Lysozyme at 2.1-a Resolution - Variability in Hydration Shell and Its Structural Consequences. J Biol Chem *265*, 16126-16131.

Kundrot, C.E., and Richards, F.M. (1987a). Crystal-Structure of Hen Egg-White Lysozyme at a Hydrostatic-Pressure of 1000 Atmospheres. J Mol Biol *193*, 157-170.

Kundrot, C.E., and Richards, F.M. (1987b). Lysozyme - Structure at 1000 Atm and Volume Fluctuations at 1 Atm. Protein Eng *1*, 231-231.

Kurinov, I.V., and Harrison, R.W. (1995). The Influence of Temperature on Lysozyme Crystals - Structure and Dynamics of Protein and Water. Acta Crystallogr D *51*, 98-109.

Lim, K., Nadarajah, A., Forsythe, E.L., and Pusey, M.L. (1998). Locations of bromide ions in tetragonal lysozyme crystals. Acta Crystallogr D *54*, 899-904.

Madhusudan, Kodandapani, R., and Vijayan, M. (1993). Protein Hydration and Water-Structure - X-Ray-Analysis of a Closely Packed Protein Crystal with Very Low Solvent Content. Acta Crystallogr D *49*, 234-245.

Nagendra, H.G., Sudarsanakumar, C., and Vijayan, M. (1996). An X-ray analysis of native monoclinic lysozyme. A case study on the reliability of refined protein structures and a comparison with the low-humidity form in relation to mobility and enzyme action. Acta Crystallogr D *52*, 1067-1074.

Nagendra, H.G., Sukumar, N., and Vijayan, M. (1998). Role of water in plasticity, stability, and action of proteins: The crystal structures of lysozyme at very low levels of hydration. Proteins *32*, 229-240.

Nanao, M.H., Sheldrick, G.M., and Ravelli, R.B.G. (2005). Improving radiation-damage substructures for RIP. Acta Crystallogr D *61*, 1227-1237.

Naresh, M.D., Jaimohan, S.M., and Kumar, V.V. (To be Published). High resolution X-ray crystal structure of tetragonal hen egg white lysozyme.

Naresh, M.D., Subramanian, V., Jaimohan, S.M., Rajaram, A., Arumugam, V., Usha, R., and Mandal, A.B. (To be published). Crystal structure analysis of hen egg white lysozyme grown by capillary method.

Oki, H., Matsuura, Y., Komatsu, H., and Chernov, A.A. (1999). Refined structure of orthorhombic lysozyme crystallized at high temperature: correlation between morphology and intermolecular contacts. Acta Crystallogr D *55*, 114-121.

Prange, T., Schiltz, M., Pernot, L., Colloc'h, N., Longhi, S., Bourguet, W., and Fourme, R. (1998). Exploring hydrophobic sites in proteins with xenon or krypton. Proteins-Structure Function and Genetics *30*, 61-73.

Ramanadham, M., Sieker, L.C., and Jensen, L.H. (1990). Refinement of Triclinic Lysozyme .2. The Method of Stereochemically Restrained Least-Squares. Acta Crystallogr B *46*, 63-69.

Ramin, M., Shepard, W., Fourme, R., and Kahn, R. (1999). Multiwavelength anomalous solvent contrast (MASC): derivation of envelope structure-factor amplitudes and comparison with model values. Acta Crystallogr D *55*, 157-167.

Rao, S.T., and Sundaralingam, M. (1996). Studies of monoclinic hen egg-white lysozyme .4. X-ray refinement at 1.8 angstrom resolution and a comparison of the variable regions in the polymorphic forms. Acta Crystallogr D *52*, 170-175.

Retailleau, P., and Prange, T. (2003). Phasing power at the K absorption edge of organic arsenic. Acta Crystallogr D *59*, 887-896.

Sauter, C., Otalora, F., Gavira, J.A., Vidal, O., Giege, R., and Garcia-Ruiz, J.M. (2001). Structure of tetragonal hen egg-white lysozyme at 0.94 angstrom from crystals grown by the counter-diffusion method. Acta Crystallogr D *57*, 1119-1126.

Steinrauf, L.K. (1998). Structures of monoclinic lysozyme iodide at 1.6 angstrom and of triclinic lysozyme nitrate at 1.1 angstrom. Acta Crystallogr D *54*, 767-779.

Takeda, K., Miyatake, H., Park, S.Y., Kawamoto, M., Kamiya, N., and Miki, K. (2004). Multi-wavelength anomalous diffraction method for I and Xe atoms using ultra-high-energy X-rays from SPring-8. J Appl Crystallogr *37*, 925-933.

Vaney, M.C., Broutin, I., Retailleau, P., Douangamath, A., Lafont, S., Hamiaux, C., Prange, T., Ducruix, A., and Ries-Kautt, M. (2001). Structural effects of monovalent anions on polymorphic lysozyme crystals. Acta Crystallogr D *57*, 929-940.

Vaney, M.C., Maignan, S., RiesKautt, M., and Ducruix, A. (1996). High-resolution structure (1.33 angstrom) of a HEW lysozyme tetragonal crystal grown in the APCF apparatus. Data and structural comparison with a crystal grown under microgravity from SpaceHab-01 mission. Acta Crystallogr D *52*, 505-517.

Vernede, X., Lavault, B., Ohana, J., Nurizzo, D., Joly, J., Jacquamet, L., Felisaz, F., Cipriani, F., and Bourgeois, D. (2006). UV laser-excited fluorescence as a tool for the visualization of protein crystals mounted in loops. Acta Crystallogr D *62*, 253-261.

Walsh, M.A., Schneider, T.R., Sieker, L.C., Dauter, Z., Lamzin, V.S., and Wilson, K.S. (1998). Refinement of triclinic hen egg-white lysozyme at atomic resolution. Acta Crystallogr D *54*, 522-546.

Wang, J.W., Dauter, M., Alkire, R., Joachimiak, A., and Dauter, Z. (2007). Triclinic lysozyme at 0.65 angstrom resolution. Acta Crystallogr D *63*, 1254-1268.

Weiss, M.S., Palm, G.J., and Hilgenfeld, R. (2000). Crystallization, structure solution and refinement of hen egg-white lysozyme at pH 8.0 in the presence of MPD. Acta Crystallogr D *56*, 952-958.

Wine, Y., Cohen-Hadar, N., Freeman, A., and Frolow, F. (2007). Elucidation of the mechanism and end products of glutaraldehyde crosslinking reaction by X-ray structure analysis. Biotechnol Bioeng *98*, 711-718.

Young, A.C.M., Dewan, J.C., Nave, C., and Tilton, R.F. (1993). Comparison of Radiation-Induced Decay and Structure Refinement from X-Ray Data Collected from Lysozyme Crystals at Low and Ambient-Temperatures. J Appl Crystallogr *26*, 309-319.
